# Sex-Dependent Effects of the Microbiome on Foraging and Locomotion in *Drosophila suzukii*

**DOI:** 10.3389/fmicb.2021.656406

**Published:** 2021-05-10

**Authors:** Runhang Shu, Daniel A. Hahn, Edouard Jurkevitch, Oscar E. Liburd, Boaz Yuval, Adam Chun-Nin Wong

**Affiliations:** ^1^Entomology and Nematology Department, University of Florida, Gainesville, FL, United States; ^2^UF Genetics Institute, University of Florida, Gainesville, FL, United States; ^3^Department of Plant Pathology and Microbiology, Faculty of Agriculture, Food and Environment, The Hebrew University of Jerusalem, Rehovot, Israel; ^4^Department of Entomology, Faculty of Agriculture, Food and Environment, The Hebrew University of Jerusalem, Rehovot, Israel

**Keywords:** microbiome, *Drosophila*, foraging, sex differences, locomotion, oviposition

## Abstract

There is growing evidence that symbiotic microbes can influence multiple nutrition-related behaviors of their hosts, including locomotion, feeding, and foraging. However, how the microbiome affects nutrition-related behavior is largely unknown. Here, we demonstrate clear sexual dimorphism in how the microbiome affects foraging behavior of a frugivorous fruit fly, *Drosophila suzukii.* Female flies deprived of their microbiome (axenic) were consistently less active in foraging on fruits than their conventional counterparts, even though they were more susceptible to starvation and starvation-induced locomotion was notably more elevated in axenic than conventional females. Such behavioral change was not observed in male flies. The lag of axenic female flies but not male flies to forage on fruits is associated with lower oviposition by axenic flies, and mirrored by reduced food seeking observed in virgin females when compared to mated, gravid females. In contrast to foraging intensity being highly dependent on the microbiome, conventional and axenic flies of both sexes showed relatively consistent and similar fruit preferences in foraging and oviposition, with raspberries being preferred among the fruits tested. Collectively, this work highlights a clear sex-specific effect of the microbiome on foraging and locomotion behaviors in flies, an important first step toward identifying specific mechanisms that may drive the modulation of insect behavior by interactions between the host, the microbiome, and food.

## Introduction

Food seeking and selection are crucial for the survival, growth, and reproduction of animals. The motivation to seek food and foraging preferences toward particular food sources involve complex integration of intrinsic (e.g., host physiological status and chemosensory perception) and extrinsic (e.g., food nutrient content and chemistry) signals. In most animals, both males and females can adjust their foraging behavior to achieve their nutritional goals and to avoid harmful components, often through the same behavioral and neurophysiological adaptations. Examples include increasing locomotion when starved to promote food searching and acquisition (Zhao et al., 2011; Yu et al., 2016), and the ability to sense or differentiate potential food sources based on volatile cues released by the food as well as cues released by associated microbes (Becher et al., 2012; Stensmyr et al., 2012; Martini et al., 2014; Karageorgi et al., 2017; Goldberg et al., 2019; Kim et al., 2019). However, males and females are distinct in their foraging motivation and reproductive investments. For example, in many oviparous insects, females make foraging decisions to fulfill both their own nutritional needs (feeding) as well as those of their offspring (oviposition). Females also allocate a large amount of energy and resources to oogenesis, requiring significant nutrient intake from the diet (Simmons and Bradley, 1997; Terashima et al., 2005; Schultzhaus and Carney, 2017). These male and female specific differences in nutritional needs are likely to drive sex-specific patterns of foraging and diet selection behaviors (Lihoreau et al., 2016; Ehl et al., 2018; Roswell et al., 2019).

Symbiosis with microbes is an important intrinsic component of animal nutrition and physiology. Contributions of the gut microbiome to a host can vary from the digestion of dietary substrates to provisioning of essential micronutrients (Larsbrink et al., 2014; Wong et al., 2014; Kovatcheva-Datchary et al., 2015; Hu et al., 2018) among other functions, which can ultimately affect host feeding and diet selection behavior (Alcock et al., 2014; Akami et al., 2019). Specifically, in the model fly *Drosophila melanogaster*, symbiotic gut bacteria can influence host foraging by priming host olfactory-guided preferences toward specific bacteria on food (Wong et al., 2017; Qiao et al., 2019), and feeding by regulating host appetite toward specific food macronutrients (Leitão-Gonçalves et al., 2017; Wong et al., 2017). Other behaviors directly linked to foraging, especially locomotor activity, have also been shown to be modulated by the gut microbiome (Schretter et al., 2018). This emerging evidence supports the notion that symbiotic microbes are an integral part of the behavioral aspects of food-seeking and acquisition. However, how the microbiome interacts with host sex-specific differences in physiology and metabolic needs for reproduction to bring about changes in behavior is unclear. Additionally, the majority of studies on foraging preference and food selection have been conducted using semi- or fully defined artificial diets. The influence of the gut microbiome on host foraging toward more natural food sources remains underexplored.

*Drosophila suzukii* (Matsumura), a close relative of *D. melanogaster*, is a significant agricultural pest with a broad host range that can infest a large variety of small, soft-skinned fruits (Hauser, 2011; Walsh et al., 2011; Asplen et al., 2015). These flies have evolved a serrated ovipositor, unique among related *Drosophila*, to lay eggs inside ripening fruits where larvae feed and develop (Hickner et al., 2016; Cloonan et al., 2018). Like *D. melanogaster*, the *D. suzukii* microbiome is dominated by a few bacterial genera, and the composition can vary significantly by geographical location and across diets (Chandler et al., 2014; Vacchini et al., 2017; Bing et al., 2018; Fountain et al., 2018; Jiménez-Padilla et al., 2020). Research using axenic *D. suzukii* generated in the laboratory has demonstrated the microbiome is essential for *D. suzukii* development on fruit (strawberry and blueberry)-based diets (Bing et al., 2018). Given the known fruit hosts and the importance of the gut microbiome in host nutrition and developmental success, *D. suzukii* can serve as a tractable model to study the relationship between the gut microbiome and foraging behavior.

In this study, we characterize the role of the gut microbiome in host foraging and locomotion using *D. suzukii* as a model. By quantifying the effects of the microbiome on flies’ overall food-seeking and host preference in both sexes (using five different fruits that are considered their natural food sources), we have made several significant discoveries. First, we reveal a strong sex difference in microbiome-mediated effects on fly foraging. Axenic females had lower food-seeking activity than conventional females, even though they were more susceptible to mortality by starvation, and starvation-induced locomotor hyperactivity was exacerbated in axenic females. Yet, we did not observe the same microbiome effects in male flies. Further, we demonstrate that female flies’ food seeking is strongly associated with egg production, by showing that axenic females laid significantly fewer eggs than conventional females, similar to virgins who also exhibited lower foraging activity than gravid females. Finally, we show that conventional and axenic flies of both sexes share similar fruit preferences; in females, their foraging and oviposition preferences toward the different fruits are tightly coupled. Altogether, our study provides novel evidence for sex-dependent effects of the microbiome on foraging and locomotion in *D. suzukii*. Sex-specific effects of the microbiome on behavior are likely prevalent across *Drosophila* species and other insects, given the evidence suggesting a significant role of microbial symbionts in insect oogenesis.

## Materials and Methods

### Fly Husbandry

Wild *D. suzukii* were collected from blackberries grown in Hawthorne Florida (29 °35′17″ N 82° 5′ 2″ W) in August 2017. The population was subsequently raised on Formula 4-24^®^ Instant *Drosophila* Medium (Carolina Biological Supply Company) supplemented with 2.5% brewer’s yeast (MP Biomedical) in the laboratory at 24°C, 64% RH, 16:8 L:D cycle. Fruit-based diets were prepared using raspberries (Driscoll’s Inc.), nectarines (PLU code: 4378, GEOFRUT Inc.), strawberries (Driscoll’s Inc.), grapes (PLU code: 4023, Ahold Inc.), and blueberries (Driscoll’s Inc.) purchased from grocery stores. Intact fruits and pitted nectarines were washed with deionized water and then macerated separately in a blender, followed by adding a solution of deionized water (13.7%), agar (0.6%), and Tegosept (0.15%), then dispensed in 50 ml bottles (VWR, United States).

### Generation of Axenic Flies

*Drosophila suzukii* mated females were allowed to lay eggs on the Instant *Drosophila* Medium overnight. Eggs were then collected in a mesh basket (2.54 cm diameter, Genesee Scientific, United States) using paintbrushes. Eggs were soaked in 0.01M sterile phosphate-buffered saline (PBS) to avoid dehydration. Axenic flies were generated using an established procedure (Ridley et al., 2013). Briefly, mesh baskets containing the eggs were soaked in 0.6% hypochlorite for 2.5 min two times. After dechorionation, embryos were rinsed three times with sterile deionized water and then placed onto fruit-based diets or autoclaved Instant *Drosophila* Medium. These steps were performed in a biosafety level II cabinet (NuAire, United States) with aseptic techniques. Successful elimination of the fly microbiota was confirmed by plating homogenates of fly adults onto MRS medium (VWR, United States).

### Collection of Virgin Flies

*Drosophila suzukii* flies emerged within 18 h were anesthetized on a *Drosophila* Flypad (Genesee Scientific, United States) using CO_2_ under a stereomicroscope and the virgin females were identified based on the presence of meconium on the ventral abdomen as well as their distinct ovipositors. Virgin females were then transferred onto autoclaved Instant *Drosophila* Medium for 7 days before conducting the foraging assay.

### Foraging and Oviposition Assays

*Drosophila suzukii* adult foraging assays were performed in transparent plastic arenas (350 × 260 × 150 mm) containing food patches made of 5 g mashed fruits loaded into open lids (25 mm diameter and 10 mm depth) and arranged in a randomized, circular array (Supplementary Figure 1). Groups of ten 5–10-day-old female or male flies were food-deprived for 15 h (provided with water), chilled on ice in microfuge tubes, before being placed at the center of the arena with the tube cap opened. Each arena was used as a biological replicate. A total number of 72 arenas were set up in the entire experiment (Conventional females, *N* = 26; Axenic females, *N* = 14; Conventional males, *N* = 17; Axenic males, *N* = 22). The number of flies on each fruit was scored at each of three-time points 7, 12, and 24 h after introduction to the arena. The number of eggs on each fruit was counted under a stereomicroscope after the 24 h-foraging assay. All fruits were purchased from grocery stores on the same day or a day before the assay. Because the fruits may vary across different batches, all four treatments (age-matched conventional female, axenic female, conventional male, and axenic male) with at least four replicates were set up on the same day. Data were aggregated from assays performed over three separate days, and day was modeled as a random factor.

### Locomotion Assays

*Drosophila suzukii* adult locomotion assays were performed in 9 cm diameter and 0.5 cm depth sterile Petri dishes that allowed free walking movement but restricted flight. Groups of eight 5–10-day-old conventional or axenic flies that had either been given open access to food or had been food-deprived for 15 h (provided with water) were placed into Petri dishes. Locomotion behaviors of flies were filmed in real-time using GigE cameras acA1300-60gc (Basler AG, Germany) for seven consecutive trials of 1 h duration from 12PM to 7PM on a laboratory bench under constant light condition and 23°C ambient temperature throughout the experiments. Each Petri dish contained eight flies that were tracked individually. Each fly in the assay was considered a replicate. For each experiment, four cameras were set up for four different groups of flies (female/male; conventional/axenic; and fed/starved), and each group was repeated once. Video footage was processed and analyzed in EthoVision XT 15 software (Noldus, Netherlands). Slightly modified from a previous study (Schretter et al., 2018), we set 0.3 mm/s and 0.1 mm/s as the threshold walking speeds for characterizing the start and stop of movement of the flies, respectively. The LOWESS (Locally Weighted Scatterplot Smoothing) method was applied to reduce the tracking noise and the small movements of the fly (“body wobble”).

### Starvation Resistance Assay

Five–ten-day-old conventional or axenic flies were sorted into same-sex groups of 15–20 individuals on ice and placed onto vials provided with 10 ml 2% agar. The number of survivors was monitored twice daily until all flies were dead. Each group of flies was replicated three times in one experiment.

### Statistical Analysis

All analyses were performed using the statistical computing environment R (version 3.5.1). Foraging assay data were analyzed by fitting either a generalized linear mixed model (GLMM) or a linear mixed model with random effects accounting for the experimental arenas and experiment days. The total numbers of foraging flies in each arena at each time point were modeled as a function of foraging time, microbiome status, sex, and their interactions using the “lmer” function. The numbers of flies counted on each fruit at each time point were modeled as a function of fruit types, foraging time, microbiome status, sex, and their interactions using the “glmer” function with Poisson distribution in R package lme4 (Bates et al., 2015). Analysis of variance for the model objects was conducted using Wald chi-square test implemented in R package car (Fox et al., 2020). Inferential statistics for all pairwise comparisons were based on 95% confidence interval of estimated marginal means (EMMs) with Kenward-Roger adjusted degrees of freedom followed by Bonferroni correction. The numbers of eggs (log-transformed) laid on each fruit were modeled as a function of fruit type and microbiome status, and their interactions. Mann-Whitney *U* tests were applied to compare the total number of eggs in each arena laid by the conventional fly population and the axenic fly population. For locomotion data, pairwise comparisons between conventional and axenic flies or between fed and starved flies were analyzed with two-sample *t*-tests after meeting the normality assumption with the Shapiro–Wilk test. Cox proportional hazards regression models and pairwise log-rank tests implemented in the R package “survival” and “survminer” were used to analyze fly mortality under starvation (Kassambara et al., 2020). All plots were generated using the R package ggplot2 (Wickham et al., 2020).

## Results

### Food-Seeking Is Reduced in Axenic Female, but Not Male *Drosophila suzukii*

**FIGURE 1:**
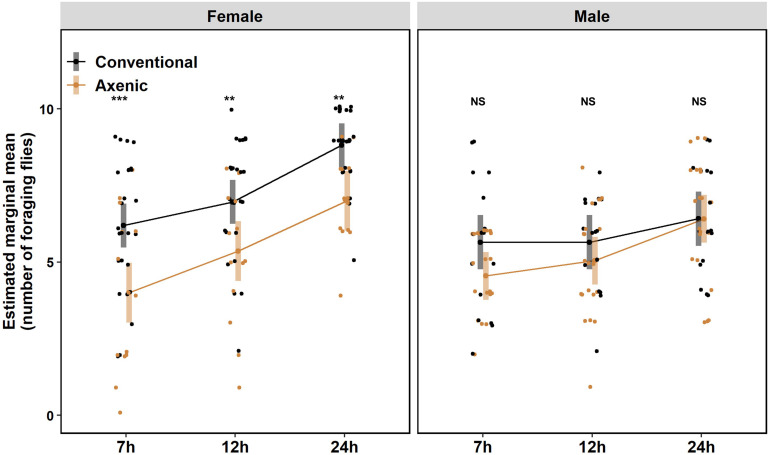
Foraging of adult *Drosophila suzukii* on fruit-based foods. Conventional females, *N* = 26; Axenic females, *N* = 14; Conventional males, *N* = 17; Axenic males, *N* = 22 (*N* indicates the number of arenas). The numbers of flies foraging on food (foraging flies) at 7, 12, and 24 h were scored. The data were analyzed by fitting a linear mixed effects model (LMM) with time, microbiome status, and sex as fixed effects (and their interactions tested), while arenas and days were accounted for as random effects. Error bars represent estimated marginal means (EMMs) with 95% confidence interval. The observed number of foraging flies in each arena was represented in dots. Statistical significance between conventional and axenic flies is indicated with ^*^P < 0.05, ^**^P < 0.01, ^***^P < 0.001. NS represents no statistical significance (*P* ≥ 0.05). *P*-values were adjusted by Bonferroni correction.

To examine the microbiome’s impact on *D. suzukii* foraging, we adopted an adult foraging assay as previously described (Wong et al., 2017). Groups of ten flies of only one sex were placed in arenas provided with five different open choices of fruits that are considered their natural hosts (Supplementary Figure 1). Both conventional (flies bearing a full microbiome) and axenic (microbiome-free) flies were tested. The total numbers of flies foraging across all five fruits increased from hour 7 to hour 24 after introduction to the arena for both axenic and conventional females, but not for either treatment in males (time:sex, *P* = 0.009, Wald, Table 1). Notably, the influence of the microbiome on flies’ food-seeking varied by sex (microbiome:sex, *P* = 0.02, Wald, Table 1). A greater number of female flies with an intact microbiome were observed foraging than axenic flies at all three time points of observation (7 h, *P* < 0.001; 12 h, *P* = 0.01; 24 h, *P* = 0.004, Bonferroni adjusted) (Figure 1), but the microbiome had a limited effect on male food seeking (7 h, *P* = 0.07; 12 h, *P* = 0.33; 24 h, *P* = 0.99, Bonferroni adjusted) (Figure 1). We discuss time spent foraging across different fruits in section “The microbiome has subtle effects on foraging and oviposition preferences toward different fruits” below.

**TABLE 1:** Summary of the GLMM/LMM Wald chi-square tests outputs.

Food-seeking: Numbers of foraging flies in response to time, sex, and microbiome status (Figure 1).			

**Fixed effects**	**Chisq**	**Df**	**Pr(>Chisq)**

Time	76.37586	2	2.60E-17
Microbiome	11.45179	1	0.000714
Sex	3.554124	1	0.059398
Time:microbiome	2.400064	2	0.301185
Time:sex	9.377775	2	0.009197
Microbiome:sex	5.724997	1	0.016725
Time:microbiome:sex	0.771933	2	0.679793

Oviposition preference: The number of eggs (log-transformed) in response to fruits and microbiome status (Figure 4).

**Fixed effects**	**Chisq**	**Df**	**Pr(>Chisq)**

Fruit	56.42379	4	1.63E-11
Microbiome	17.29838	1	3.19E-05
Fruit:microbiome	6.531558	4	0.162813

Fruit preference: Fruit choice in response to time, fruits, sex, and microbiome status (Figure 4).

**Fixed effects**	**Chisq**	**Df**	**Pr(>Chisq)**

Time	28.43574	2	6.69E-07
Fruits	210.3089	4	2.28E-44
Microbiome	8.975631	1	0.002736
Sex	3.605242	1	0.057598
Time:fruits	9.749983	8	0.283014
Time:microbiome	2.03618	2	0.361284
Fruits:microbiome	2.610014	4	0.62505
Time:sex	2.87031	2	0.238078
Fruits:sex	5.548641	4	0.235487
Microbiome:sex	4.322436	1	0.037613
Time:fruits:microbiome	24.03812	8	0.002258
Time:fruits:sex	9.106219	8	0.333415
Time:microbiome:sex	0.268544	2	0.874352
Fruits:microbiome:sex	2.778305	4	0.595583
Time:fruits:microbiome:sex	7.6383	8	0.469575

### Axenic Female *D. suzukii* Are More Sensitive to Starvation

Foraging behavior is modulated by nutritional status (Toth et al., 2005; Pradhan et al., 2019). Accordingly, the lower numbers of axenic females observed on food compared to conventional females led us to hypothesize that axenic female *D. suzukii* have reduced hunger or appetite and would thus potentially be less sensitive to starvation. Contrary to our expectation, axenic female *D. suzukii* were more susceptible to death by starvation than conventional females (*P* = 7.4 × 10^–5^, log-rank) (Supplementary Figure 2). Male flies were generally less starvation-resistant than female flies (sex, *P* = 4.4 × 10^–10^, Cox regression), but no difference was observed between conventional and axenic males (*P* = 1, log-rank) (Supplementary Figure 2).

**FIGURE 2:**
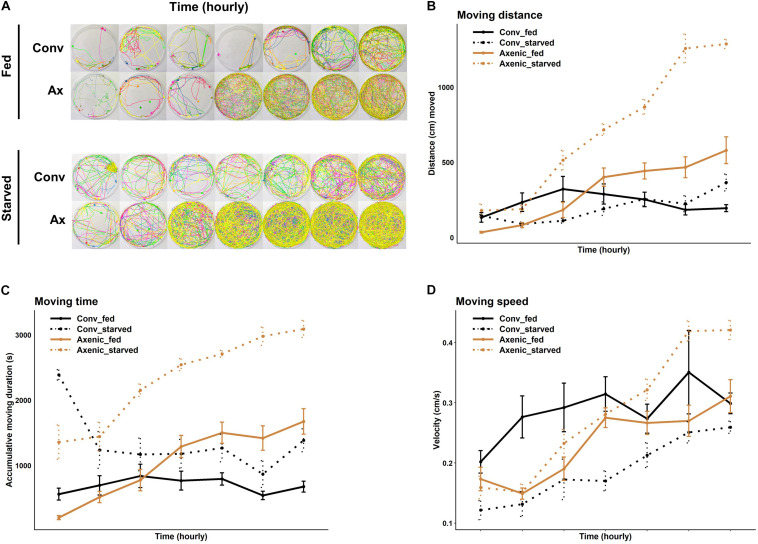
Effects of the microbiome on locomotor activities of fed or starved female *Drosophila suzukii*. **(A)** Movement profiles of groups of eight conventional (Conv) and axenic (Ax) flies in the locomotion assay at 1 h intervals. **(B)** Hourly moving distance, **(C)** moving time, and **(D)** moving speed of fed or starved conventional flies (black lines) and axenic flies (golden lines) during the 7 h. **(B–D)** Fed conventional flies, *n* = 16; starved conventional flies, *n* = 16; fed axenic flies, *n* = 16; starved axenic flies, *n* = 16. *n* indicates the number of flies tracked. The error bars represent the means ± SEM (standard error of the means). All pairwise comparisons were analyzed with two-sample *t*-tests after meeting the normality assumption with Shapiro–Wilk test. Full statistical details are in Supplementary Table 2.

Starvation has been shown to increase animal locomotor activity (Lee and Park, 2004; Isabel et al., 2005; Dietrich et al., 2015; Yang et al., 2015; Yu et al., 2016), presumably to facilitate exploration of the environment and promote the chance of locating food. Interestingly, a recent study on *D. melanogaster* showed axenic females were hyperactive compared to conventional females, but male flies were not tested, and the effect of starvation was unknown (Schretter et al., 2018). Therefore, we compared locomotion between axenic and conventional *D. suzukii* of both sexes under fed and starved conditions. Consistent with the observation in *D. melanogaster*, axenic *D. suzukii* females were hyperactive compared to conventional females (Figure 2A). Fed axenic females traveled ∼2.6 times further (*P* = 3.5 × 10^–7^, Bonferroni adjusted) and walked for ∼2.4 times longer (*P* = 1.4 × 10^–8^, Bonferroni adjusted) than fed conventional females (Figures 2B,C).

Starvation-induced locomotor hyperactivity was more pronounced in axenic females than conventional females. Both distance moved and duration of movement were further increased in axenic females under starvation in the 7-h window, by 129% and 121%, respectively. In conventional females, starvation increased the duration of movement (*P* = 2.5 × 10^–10^, Bonferroni adjusted) but not the distance moved (*P* = 0.4, Bonferroni adjusted), owing to decreased walking speed in starved flies (Figures 2A–D and see Supplementary Table 2 for statistical details).

Male flies exhibited different locomotion patterns from female flies in response to microbiome elimination and starvation. At the beginning of the assay, starved males displayed significantly greater moving distance and moving duration than fed males, regardless of the microbiome status (Supplementary Figure 3A). Interestingly, toward the later time points (from 4 to 7 h), the locomotion of conventional fed males was significantly elevated, with up to a 7.6 times increase in moving distance and five times increase in moving duration (see Supplementary Figures 3B,C and Supplementary Table 2 for statistical details).

### Egg-Laying on Fruits Is Dramatically Reduced in Axenic *D. suzukii*

**FIGURE 3:**
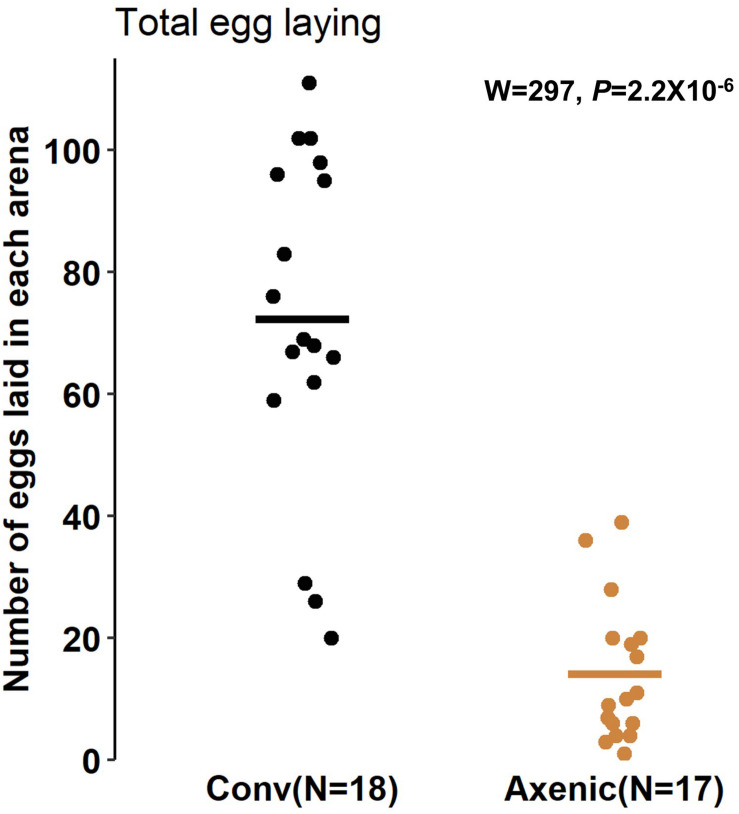
Oviposition of conventional and axenic *Drosophila suzukii*. The total number of eggs laid on the fruits after 24 h in the arenas (Mann–Whitney *U* test).

The reduced food seeking in axenic *D. suzukii* occurs only in females, seemingly contradicting the higher sensitivity of females to starvation, both in starvation resistance and starvation-induced locomotor response. Based on these observations, we hypothesized that the female-specific microbiome effect on foraging might be associated with oviposition, because females forage for fruits to lay eggs in addition to their own consumption. In fact, we observed that conventional female populations laid over five times more eggs than the axenic female populations 24 h after being placed in the arenas. The average numbers of eggs laid by the conventional females were 76 ± 7.3 (SEM), compared to 14 ± 2.8 (SEM) laid by axenic females in each arena (W = 297, *P* = 2.2 × 10^–6^, Mann-Whitney *U* test) (Figure 3). To further elucidate the relationship between food seeking and egg laying, we also compared foraging between virgin (no egg laying) and gravid flies. As expected, conventional virgin flies were less active in seeking food than conventional gravid flies, but the difference was only significant at the early time point (7 h, *P* = 0.027, Bonferroni adjusted) (Supplementary Figure 4). Also, no difference in food seeking between virgin and gravid flies was observed when they were axenic (7 h, *P* = 0.24; 12 h, *P* = 0.17; 24 h, *P* = 0.60, Bonferroni adjusted) (see Supplementary Figure 4 and Supplementary Table 1 for statistical details). Together, our data point to reduce egg production and laying as a possible mechanism for reduced foraging behavior in axenic flies, but additional factors are likely involved in the microbiome-dependent effect on female food seeking.

### The Microbiome Has Subtle Effects on *D. suzukii* Foraging and Oviposition Preferences Toward Different Fruits

**FIGURE 4:**
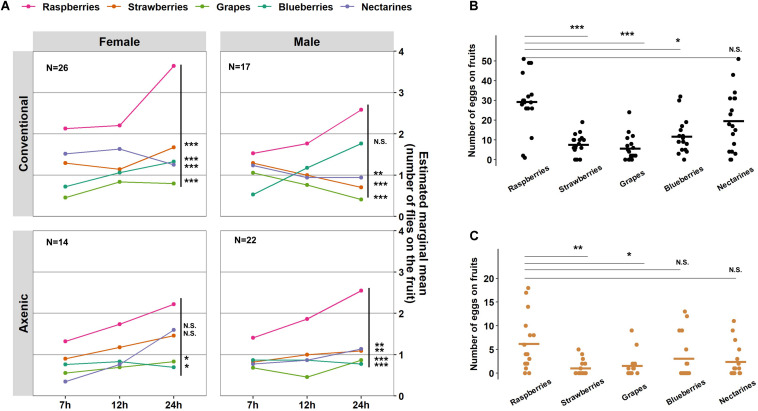
Effects of the microbiome on *Drosophila suzukii* fruit preferences and oviposition preference. **(A)** A generalized linear mixed model (GLMM; Poisson) was applied to analyze the number of flies on each fruit in response to time, microbiome status, and sex as fixed effects (and their interactions tested), while arenas and days were accounted for as random effects. The number of eggs laid on each fruit by **(B)** the conventional fly populations or **(C)** the axenic fly populations. Statistical significance between raspberries and the other four fruits is indicated with ^*^P < 0.05, ^**^P < 0.01, ^***^P < 0.001. NS represents no statistical significance (*P* ≥ 0.05). The crossbars represent mean values. *N* indicates the number of arenas with each containing ten *D. suzukii* adults. *P*-values were adjusted by Bonferroni correction.

In addition to overall food seeking and total egg production, we examined the microbiome’s impact on *D. suzukii* fruit preferences. Conventional and axenic flies of both sexes displayed similar relative fruit preferences in foraging (fruits:sex, *P* = 0.24; fruit:microbiome, *P* = 0.63; fruit:microbiome:sex, *P* = 0.60, Wald; Table 1). In the conventional populations, female flies preferred foraging on raspberries over strawberries (*P* = 0.0002, Bonferroni adjusted), blueberries (*P* < 0.0001, Bonferroni adjusted), nectarines (*P* < 0.0001, Bonferroni adjusted), and grapes (*Vitis vinifera*) (*P* < 0.0001, Bonferroni adjusted) (Figure 4A). Male flies were most attracted to raspberries and blueberries, followed by nectarines (*P* = 0.0053, Bonferroni adjusted), strawberries (*P* = 0.007, Bonferroni adjusted), and grapes (*P* = 0.0001, Bonferroni adjusted) (Figure 4A and see Supplementary Table 1 for statistical details). Subtle differences in fruit preferences were observed in axenic flies. Specifically, a reduced preference for blueberries was detected in axenic males, and the preference for raspberries was less pronounced in axenic females than in conventional females.

Aligning with the foraging preference, conventional females preferred laying eggs on raspberries, distributing 41.9% of the eggs on raspberries, followed by 25.7% on nectarines, 15.3% on blueberries (*P* = 0.02, Bonferroni adjusted), 9.9% on strawberries (*P* < 0.0001, Bonferroni adjusted), and 7.3% on grapes (*P* < 0.0001, Bonferroni adjusted) (Figure 4B). Although axenic flies laid significantly fewer eggs than conventional flies, microbiome’s effect on oviposition preference was not significant (fruit:microbiome, *P* = 0.16, Wald, Table 1). The relative proportion of eggs laid on raspberries by axenic females was 43.8%, followed by 21.7% on blueberries, 16.7% on nectarines, 10.8% on grapes (*P* = 0.01, Bonferroni adjusted), and 7.1% on strawberries (*P* = 0.001, Bonferroni adjusted) (Figure 4C). Together, our results suggest that *D. suzukii* female foraging preference generally aligns with oviposition preferences, regardless of the flies’ microbiome status.

Given that female *D. suzukii* distributed their eggs across the different fruits, it raises the question of whether flies developing on different fruits in early life may differ in their later-life foraging preferences. To test this, we raised the flies on three different fruits (raspberry, strawberry, and nectarine) for one (F1) or five generations (F5), then subjected the flies to the foraging assays offered with the different fruits. Our results suggest that the foraging fruit preferences of *D. suzukii* are not dependent on their diet history. Regardless of the fruit they were raised on, the flies maintained the strongest preference toward raspberries and a similar order of preference on the other fruits. The results are consistent regardless of whether the flies were raised on those fruits for one or five generations (Supplementary Figure 5 and Supplementary Table 3).

## Discussion

Using a non-model *Drosophila* system, our experiments show significant effects of the microbiome on three discrete but connected host behaviors: foraging, oviposition, and locomotion. These findings join a growing body of literature demonstrating that the gut microbiome contributes to individual patterns of animal behavior, by way of modulating internal physiological processes and the nervous system (Ben-Ami et al., 2010; Alcock et al., 2014; Mayer et al., 2015; Wong et al., 2017; Akami et al., 2019; Morimoto et al., 2019; Hosokawa and Fukatsu, 2020). Our results show that food seeking in female, but not male *D. suzukii*, is influenced by the fly’s microbiome. Lower food seeking in axenic females is associated with reduced egg production but conflicted with the flies’ higher locomotor response and susceptibility to starvation, suggesting that the flies might prioritize foraging for oviposition sites for their progeny over their own feeding. This is also supported by the close alignment between fly foraging and oviposition preferences, with raspberries being the most preferred fruit among the five fruits tested.

Our finding of decreased egg laying in axenic *D. suzukii* is in line with previous studies on other insects showing that symbionts can promote host reproduction, including mosquitoes (Gaio et al., 2011; Coon et al., 2016), olive fruit fly (Jose et al., 2019), Queensland fruit fly (Nguyen et al., 2020), the bean bug *Riptortus pedestris* (Lee et al., 2017), and *Drosophila melanogaster* (Leitão-Gonçalves et al., 2017). Specifically, there is emerging evidence for the role of microbial symbionts in insect oogenesis. In *D. melanogaster*, the gut microbiome has been shown to contribute to oogenesis in two independent studies. Elgart et al. (2016) found that reduction in oogenesis of axenic female flies is associated with reduced transcription and enzymatic activities of *Aldh* (*Aldehyde dehydrogenase*) in the ovary. *Aldh* expression and the normal oogenesis phenotype could be restored by re-introduction of a specific *Acetobacter* gut bacterium to the flies. Gnainsky et al. (2021) suggested that specific gut bacteria provision an essential B vitamin (riboflavin) and mitochondrial co-enzymes to the host to support oogenesis. While further research is needed to elucidate the involvement of these molecular pathways in microbiome-dependent egg production in *D. suzukii*, our study is the first to discover that the microbiome-dependent effects on egg production could have an effect on the foraging behavior of the host insects.

Egg production requires nutrient acquisition and allocation (i.e., vitellogenin) to the oocytes. A recent study on *D. melanogaster* showed egg production depends on the upregulation of the pentose phosphate pathway in the germline, which in turn affects sugar feeding (Carvalho-Santos et al., 2020). Therefore, foraging signals are likely elicited or suppressed during the pre-oviposition state, corresponding to nutritional needs for egg production. In *Aedes* mosquitoes, vitellogenin synthesis in the fat body is upregulated after a blood or sugar meal, and the elevated expression of a specific vitellogenin gene (*Vg-2*) has been shown to suppress host-seeking behavior (Hansen et al., 2014; Dittmer et al., 2019). Conserved mechanisms governing the equilibrium between foraging, egg production, and egg-laying behavior may also exist in other insects. More recently, a study on Queensland fruit fly highlights transgenerational effects of the parental microbiome on offspring fecundity (Nguyen et al., 2020). Therefore, it is also plausible that the microbiome could exert long-term effects on the host fly germline that ultimately shapes their hosts’ foraging and oviposition strategies. Besides effects on food seeking and oviposition, our results demonstrate sexual dimorphism in locomotion in axenic flies. Our locomotion data corroborate recent findings in *D. melanogaster*, showing axenic females were inherently hyperactive compared to conventional females (Schretter et al., 2018). We show that starvation-induced hyperactive locomotion was exacerbated in axenic females. In contrast, the locomotion in male *D. suzukii* was less affected by the microbiome. In *D. melanogaster*, it is believed that an enzyme (xylose isomerase) encoded by a specific fly gut commensal bacterium (*Lactobacillus brevis)* can rescue fly locomotion in axenic flies to levels similar to conventional flies by modulating host sugar metabolism and possibly octopamine signaling (Schretter et al., 2018). Interestingly, *Lactobacillus brevis* was not found in our *D. suzukii* flies (data not shown), but other gut bacteria we have detected in *D. suzukii* (including *Bacillus* sp. and *Enterobacter* sp.) might encode this enzyme (Amore et al., 1989; Brat et al., 2009; Taghavi et al., 2010). Assuming the same bacteria-mediated mechanism is at play in controlling *D. suzukii* locomotion as in *D. melanogaster*, a plausible explanation is that there is a sex difference in fly behavioral output in response to the same microbiome-dependent effector(s) (or the lack of such effectors). For instance, octopamine signaling has been shown to drive aggression (Hoyer et al., 2008) and plasticity responses to endurance exercise (Sujkowski et al., 2017) specifically in male *D. melanogaster*, while for females, it can stimulate post-mating behaviors, including oviposition (Rezával et al., 2014).

Taken together, our work, using a non-model fruit fly, provides the first demonstration of a role for the microbiome in host foraging behavior associated with changes in host physiological state. The interrelationship between host-microbe symbiosis, oviposition, and foraging might be ubiquitous across insect taxa, given that the role of symbiotic microbes in insect oogenesis has been established in different insects. The knowledge of how commonly the microbiome affects foraging and oviposition behaviors opens new research avenues regarding microbiomes as key regulators of animal behavior. It could also serve as a basis for innovative strategies to control pest insects by diminishing their tendency to forage and oviposit on crops, via disruption of their microbiomes to achieve long-term physiological and behavioral changes.

## Data Availability Statement

The original contributions presented in the study are included in the article/Supplementary Material, further inquiries can be directed to the corresponding author/s.

## Author Contributions

AW and RS conceived the ideas, designed the methodology, and wrote the initial manuscript draft. RS conducted the experiments, collected the data, and led the statistical analyses. DH, EJ, OL, and BY critically reviewed the methodology and results. All authors contributed critically to the manuscript writing and gave approval for the final submission.

## Conflict of Interest

The authors declare that the research was conducted in the absence of any commercial or financial relationships that could be construed as a potential conflict of interest.
