# Supplementary material for: Sex-Dependent Effects of the Microbiome on Foraging and Locomotion in Drosophila suzukii
Source: Front Microbiol. 2021 May 10;12:656406. doi: 10.3389/fmicb.2021.656406 (PMC8141744; doi:10.3389/fmicb.2021.656406)
Supplement: Supplementary file 1 [file Data_Sheet_1.pdf]

Supplementary materials

Fig. S1

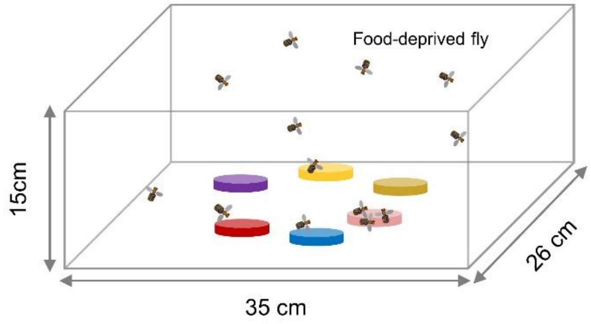

Fig. S1 Setup of the adult foraging assay.

Fig. S2

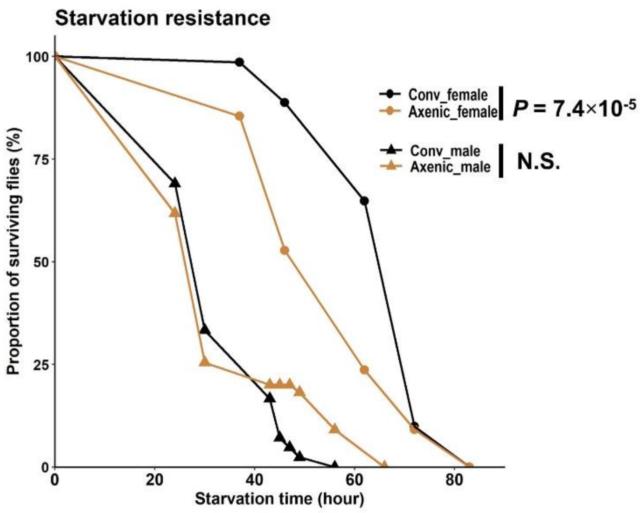

Fig. S2 Starvation resistance of conventional and axenic flies. Conventional female, n=71; Axenic female, n=55; Conventional male, n=42; Axenic male, n=55. n indicates the number of flies in the

assay. The data were analyzed by fitting a Cox proportional hazards model with time being the predictor variable in response to microbiome status and sex. Log-rank tests were applied to analyze the pairwise comparisons of conventional and axenic flies. N.S. represents no statistical significance ($P \geq 0.05$). P value is adjusted by Bonferroni correction.

Fig. S3

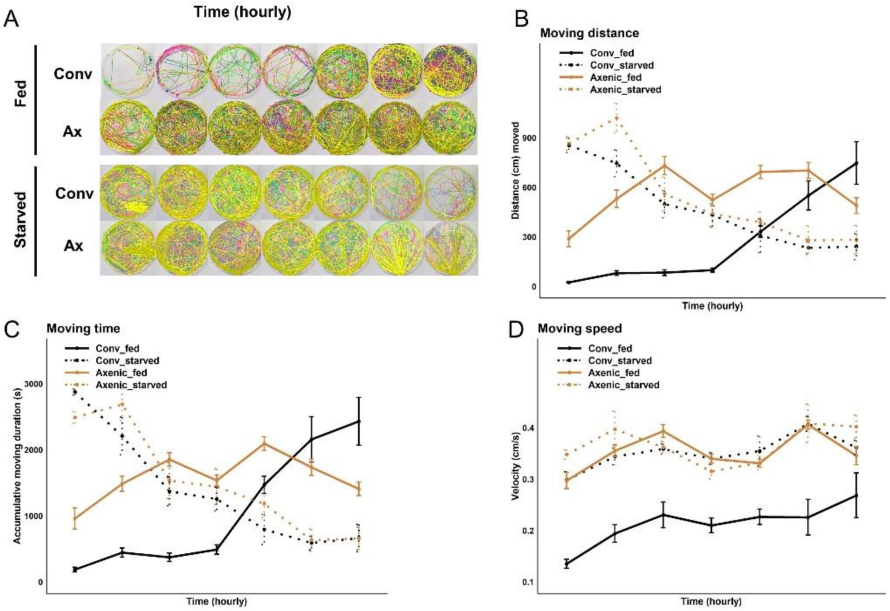

Fig. S3 Effects of the microbiome on locomotor activities of male *D. suzukii*. (A) Movement profiles of groups of eight conventional (Conv) and axenic (Ax) flies in the locomotion assay at 1h intervals. (B) Hourly moving distance, (C) moving time, and (D) moving speed of fed or starved conventional flies (black lines) and axenic flies (golden lines) during the 7 hours. (B-D) Fed conventional flies, n=8; starved conventional flies, n=8; fed axenic flies, n=8; starved-axenic flies, n=8. n indicates the number of flies tracked. The error bars represent the means ± SEM (standard error of the means). All pairwise comparisons were analyzed with two-sample t-tests after meeting the normality assumption with Shapiro-Wilk test. Full statistical details are in Table S2.

Fig. S4

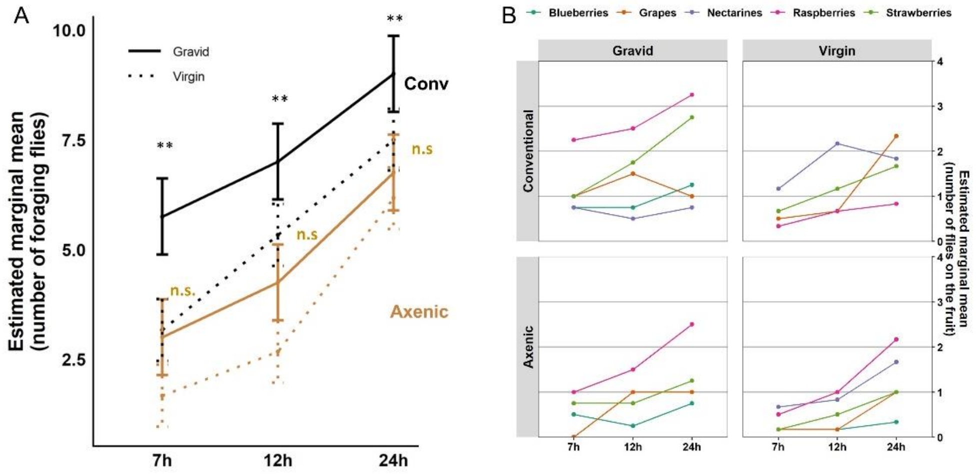

Fig. S4 Foraging of virgin and gravid female *D. suzukii*. The number of flies foraging on fruits (A) and the number of flies on each fruit (B) in each arena was fitted into a linear mixed effects model (LMM) and a generalized linear mixed effect model (GLMM: Poisson), respectively. Conventional gravid, N=4; conventional virgin, N=6; axenic gravid, N=4; axenic virgin, N=6 (N indicates the number of arenas with each containing ten *D. suzukii*). Arena was fitted as an random effect. The error bars represent Mean ± SEM (Standard Error of Mean). Full statistical details are in Table S1.

Fig. S5

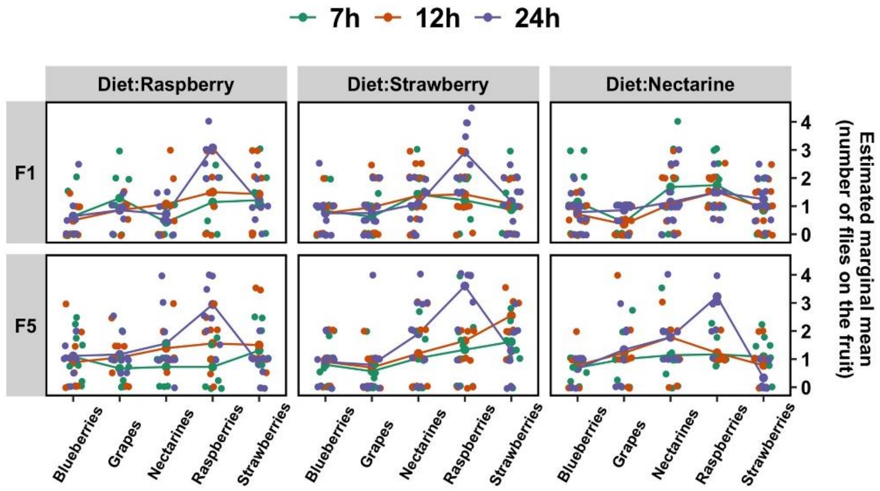

Fig. S5 Effect of dietary history on *D. suzukii* fruit preference. The fruit preferences of flies raised on different fruits (raspberries, strawberries, nectarines) for one generation (from egg to adult, F1) or for five generations (F5) were tested in the foraging assay. The data were analyzed by fitting a generalized mixed effects model with Poisson distribution. Pairwise comparisons were conducted based on Estimated Marginal Mean (EEMs) in response to diet, fly generation, fruit choice as fixed effects (and their interactions tested). Full statistical details are in Table S3.
